# Relationship Between Buddhist Belief and Suicide Risk in Chinese Persons Undergoing Methadone Maintenance Therapy for Heroin Dependence

**DOI:** 10.3389/fpsyt.2020.00414

**Published:** 2020-05-07

**Authors:** Jian-Xing Huang, Yan-Min Xu, Bao-Liang Zhong

**Affiliations:** ^1^College of Sociology and History, Fujian Normal University, Fuzhou, China; ^2^Department of Psychiatry, The Affiliated Brain Hospital of Guangzhou Medical University (Guangzhou Huiai Hospital), Guangzhou, China; ^3^Affiliated Wuhan Mental Health Center, Tongji Medical College of Huazhong University of Science & Technology, Wuhan, China

**Keywords:** Buddhist belief, suicide risk, heroin dependence, methadone maintenance treatment, association, China

## Abstract

**Background:**

In western countries, there is a negative association between religious belief and suicide risk, while in China this association is positive. Nevertheless, few data are available on the association between one specific type of religion and suicide risk, which might be different from the overall positive religion-suicide association in China. This study examined the association between Buddhist belief and suicide risk in Chinese persons receiving methadone maintenance therapy (MMT) for heroin dependence.

**Methods:**

In total, 61 Buddhist believers and 425 age, gender, and clinic frequency-matched non-religious believers were selected from a sample of patients with heroin dependence treated in three MMT clinics in Wuhan, China. The suicidality module of the Chinese version of the Mini-international Neuropsychiatric Interview 5.0 was used to assess current suicide risk. Patients' demographic and clinical characteristics were also collected. Multiple ordinary logistic regression was used to analyze the association between Buddhist belief and current suicide risk, controlling for the confounding effects of demographic and clinical factors.

**Results:**

In Chinese patients receiving MMT for heroin dependence, Buddhist believers had significantly higher levels of current suicide risk than non-religious believers (low: 45.9% vs. 24.7%, medium: 4.9% vs. 3.5%, high: 19.7% vs. 12.5%, P < 0.001). After adjusting for demographic and clinical covariates (including depressive symptoms), Buddhist belief was still significantly associated with an increase in the level of current suicide risk (OR: 2.98, P < 0.001).

**Conclusion:**

Buddhist belief is significantly associated with elevated current suicide risk in Chinese patients receiving MMT for heroin independence. In Chinese MMT clinics, patients with Buddhist belief may have a high current suicide risk and a timely psychiatric assessment and crisis intervention (when necessary) should be provided to these patients.

## Introduction

Religious beliefs can affect a person's mental health help-seeking behaviors and perceptions of mental well-being, meaning of life, pain, and psychiatric symptomatology (1–4). Despite some controversies over the positive effect of religion on mental health, in western countries, most studies have shown that religious involvement is associated with better mental health, including lower risk of depression, substance abuse, and death due to suicide (5). In general, religion can provide material, moral, emotional, and social supports to people who regularly participate in religious organizations and activities, which in turn buffer the negative effect of stressful life events, thereby maintaining/promoting mental health and reducing suicide risk (6). It is also speculated that religious beliefs could lower an individual's suicide risk by increasing the sense of coherence, because suicide can be viewed as a manifestation of the lack of social cohesion in Durkheim's theory of suicide (7, 8). Accordingly, researchers have developed some religious psychotherapies or integrated religious beliefs into psychotherapies to address patients' mental health and suicidal problems, and some empirical studies have provided promising results on the effectiveness of such treatments (9–12).

However, most evidence on the beneficial effects of religion is derived from studies conducted in Christian countries that are economically developed and generally believed to be religious (5, 13). As suggested by findings from two systematic reviews, although religion does protect against attempted and completed suicide, this effect varies depending on the cultural and religious context because both religion and suicidal behaviors are multidimensional and complex (14, 15). Due to cultural differences, it might be difficult to generalize western findings to people of less religious and non-Christian countries such as China. For example, in Chinese general hospitals, outpatients with religious beliefs are at significantly greater risk for depressive disorders than those without religious beliefs, and religious beliefs are significantly associated with higher levels of suicide intent among suicide attempters (16, 17), and, in the Chinese general population, religious beliefs are positively associated with an elevated risk of mental disorders and attempted and completed suicide (18–20). So it seems that religion is harmful for the mental health of people in China. Some Chinese researchers have ascribed this phenomenon to the weaker sense of cultural identity and belonging of religious believers, because they are a minority group in this atheist-dominated country, China, accounting for only 10% of the total population (21, 22). Nevertheless, we must be cautious to generalize the above findings in China, because an overall significant association does do not mean that all types of religion are associated with elevated risk of mental health problems and suicide and, in fact, there has been evidence showing that the risk of psychological distress and suicidal behaviors differs across believers of various religions (23). China has a variety of religions, including Buddhism, Taoism, Christianity, and Islam (22), it is therefore necessary to further examine the association between one specific religious belief and risk of mental health problems and suicidal behaviors, which may help deepen our understanding on the mechanisms underlying the religion-mental health association. Unfortunately, due to the small numbers of religious believers in previous studies, few of them did stratified analyses according to religions.

In China, heroin use remains a public health concern (24), and in 2016, there were 162,000 Chinese individuals receiving methadone maintenance therapy (MMT) for heroin dependence (25). These patients with heroin dependence, although they are undergoing MMT, are still at higher risk of completed suicide and other non-fatal suicidal behaviors (26–29). Some studies have reported a variety of factors associated with suicidal behaviors in this patient population, including female gender, a low level of education, inadequate social support, and depression (26–28). However, as far as we know, the association of religious affiliation with suicide risk has not been examined in Chinese patients undergoing MMT for heroin dependence.

The present study investigated the association between Buddhist belief and suicide risk among a sample of Chinese MMT patients. Buddhism was the religion of interest in this study, because it is the most common type of religion in China and suicide is generally contrary to Buddhism ethics (22, 30).

## Materials and Methods

### Participants

This study was a secondary data analysis of a large-scale survey, which was conducted to investigate the mental health, self-destructive behaviors, sexual life satisfaction, and quality of life among Chinese patients of three municipality-owned MMT clinics in Wuhan, China, between June 2009 and July 2010 (31–33). By using consecutive sampling method, all patients who were 20 years old or older, met DSM-IV criteria for heroin dependence, and were undergoing MMT in the three clinics were invited to participate in the study. We excluded patients with severe physical illnesses and psychotic symptoms, as well as those with diagnoses of alcohol dependence and brain organic mental disorders.

In total, 743 patients were receiving MMT at the three selected clinics. Among them, 652 met the inclusion criteria after assessment for eligibility and all participated in the study. Because 16 patients withdrew the survey and 33 patients did not complete the survey, our study finally collected complete questionnaires from 603 MMT patients: 86 with and 517 without religious belief. Proportions of men (31.7% v. 28.6%, χ2 = 0.202, P = 0.653) and mean ages (38.1 ± 7.0 v. 36.5 ± 6.8, t = 1.542, P = 0.124) did not differ significantly between the 603 completers and 49 non-completers.

Among the 86 believers, 61 were Buddhist believers. The current study was designed as a comparative study, comparing the suicide risk between Buddhist and those without religious belief (non-religious believers). Therefore, a comparison group of 425 non-religious believers was purposively selected from the 517 non-religious believers, which was age, gender, and clinic frequency-matched to the group of 61 Buddhist believers.

The study was reported according to STROBE (the Strengthening the Reporting of Observational Studies in Epidemiology Statement) guideline (34). Because this was a secondary data analysis, the association between Buddhist belief and suicide risk was not of primary interest of the primary study, we did not pre-calculate sample size required for the current analysis. Nevertheless, results of the post-hoc analysis suggested that the sample size of our study was large enough for the current analysis, because we obtained a statistical power of 0.996 given that proportions of persons at risk for suicide were 0.705 in Buddhist believers and 0.407 in non-religious believers, sample sizes were 61 for Buddhist believers and 425 for non-religious believers, and a statistical significance level was targeted at 0.05.

Written informed consent was obtained from all participants. The institutional review board of Wuhan Mental Health Center approved the study protocol before the formal survey.

### Instruments

The study used a standardized self-administered questionnaire to collect data. Trained investigators were arranged to read out the questions for patients who had difficulties in completing their questionnaires.

Demographic variables in the questionnaire included age, gender, education, marital status, and employment status.

Clinical factors included the main route of past heroin use (smoking vs. injecting), duration of past heroin use, duration of MMT, dosage of methadone (md/d), and pain. The pain intensity was assessed with the five-point Verbal Rating Scale (“Overall, how intense is your pain now?”) (35). The five response options were “1=none”, “2=mild”, “3=moderate”, “4=severe”, and “5=very severe”. In concordance with previous studies, patients who selected 3–5 were regarded as having clinically significant pain (31, 36). This single-item measure of pain has been widely in a variety of clinical and non-clinical settings with satisfactory validity for assessing pain intensity (35, 37).

Depressive symptoms were assessed with the Chinese version of Zung's Self-rating Depression Scale (SDS) (38). SDS has 20 items with each item being rated on a 4-point Likert scale from “1=a little of the time” to “4=most of the time”. The total raw score of SDS ranges from 20 to 80, with higher scores corresponding to more severe depressive symptoms. The Chinese SDS has been proved to be valid and reliable in assessing the severity of depressive symptoms for Chinese population, and a total SDS score of 40 and above indicates clinically significant depressive symptoms (39).

In this study, two standardized questions were used to determine the presence of Buddhist belief. The first question, asked of all patients, was ‘‘Do you have religious beliefs?''. Patients answering “yes” were further asked a second question: ‘‘what is your religion?'' Respondents who answered ‘Buddhism'' were classified as having Buddhist belief.

The suicidality module of the validated Chinese version of the Mini-international Neuropsychiatric Interview 5.0 was used to assess current suicide risk (40). This module includes five specific questions regarding death wish (if present, the score is 1), self-harm (if present, the score is 2), suicidal ideation (if present, the score is 6), suicide plan (if present, the score is 10), and suicide attempt (if present, the score is 10) within the past month and one question regarding lifetime suicide attempt (if present, the score is 10). Based on the total score of this module, current suicide risk of a respondent is categorized into four mutually exclusive levels: no (0 score), low (1–5 score), medium (6–9 score), and high (≥10 score).

### Statistical Analysis

SPSS software version 17.0 package (SPSS Inc., Chicago, IL, USA) was used for all analyses. Demographic and clinical variables, the presence of depression, and suicide risk between Buddhist and non-religious believers were described and compared by t-test or Chi-square test, as appropriate. The association between Buddhist belief and suicide risk was examined with multiple ordinary logistic regression that entered the level of current suicide risk as the outcome variable, Buddhist belief as the predictor, and demographic and clinical variables and depression all together as covariates. This analytic approach was used to adjust for the potential confounding effects of demographic and clinical variables and depression. Because these demographic and clinical variables and depression have been reported to be factors associated with or risk factors of suicidal behaviors among general population and patients with heroin dependence (29, 41–46), they were included as covariates in the above adjustment analysis. Odds ratios (ORs) and their 95% confidence intervals (95%CIs) were used to quantify associations between factors and the increase in suicide risk. The statistical significance level was set at P < 0.05 (two-sided).

## Results

As shown in **Table 1**, although Buddhist and non-religious believers were comparable with respect to most demographic and clinical variables, Buddhist believers were significantly more likely to have an educational level of junior high school and above, pain, and depressive symptoms (P ≤ 0.004).

**Table 1:** Characteristics and current suicide risk of Buddhist and non-religious believers among Chinese persons receiving methadone maintenance therapy (MMT) for heroin dependence.

Characteristics	Buddhist believers (n=61)	Non-religious believers (n=425)	Statistics	P
Clinic*				
* Diyi*	38 (62.3%)	280 (65.9%)		
* Hanyangweimin*	14 (23.0%)	74 (17.4%)		
* Gutian*	9 (14.8%)	71 (16.7%)	χ2 = 1.132	0.568
Gender: male*	39 (63.9%)	298 (70.1%)	χ2 = 0.959	0.327
Age (years)^#^	40.1 (7.2)	39.1 (6.0)	t=1.118	0.264
Education: junior high school and above*	34 (55.7%)	156 (36.7%)	χ2 = 8.115	0.004
Marital status: non-married*^$^	30 (49.2%)	211 (49.6%)	χ2 = 0.005	0.946
Unemployment*	27 (46.6%)	189 (46.4%)	χ2 = 0.001	0.987
Main route of heroin administration: smoking*	13 (21.3%)	68 (16.1%)	χ2 = 1.032	0.310
Duration of heroin use (years)^#^	10.8 (3.7)	10.2 (4.2)	t = 1.073	0.284
Duration of MMT (months)^#^	24.7 (10.6)	25.4 (10.5)	t = 0.452	0.652
Dosage of methadone (mg/d)^#^	68.3 (27.6)	68.8 (27.6)	t = 0.149	0.882
Pain*	49 (80.3%)	216 (50.8%)	χ2 = 18.728	<0.001
Depressive symptoms*	36 (62.1%)	145 (35.5%)	χ2 = 15.046	<0.001
Current suicide risk*				
No	18 (29.5%)	252 (59.3%)		
Low	28 (45.9%)	105 (24.7%)		
Medium	3 (4.9%)	15 (3.5%)		
High	12 (19.7%)	53 (12.5%)	χ2 = 19.622	<0.001

*Categorical variables are expressed as counts (percentages).

^#^Continuous variables are expressed as means (standard deviation).

^$”^Non-married” includes never-married, separated, cohabitating, divorced, and widowed.

MMT, methadone maintenance therapy.

Levels of current suicide risk were significantly higher in Buddhist than non-religious believers (low: 45.9% vs. 24.7%, medium: 4.9% vs. 3.5%, high: 19.7% vs. 12.5%, P < 0.001) (**Table 1**). After adjusting for demographic and clinical covariates and depression, Buddhist belief was still significantly associated with an increase in the level of current suicide risk (OR: 2.98, 95%CI: 1.66, 5.34, P < 0.001) (**Table 2**).

**Table 2:** Multiple ordinal logistic regression on the association between Buddhist belief and current suicide risk in Chinese persons receiving methadone maintenance therapy for heroin dependence, controlling for demographic and clinical factors and depressive symptoms.

Characteristics	OR(95%CI)	P
Buddhist belief (vs. non-religious)	2.98 (1.66, 5.34)	<0.001
Clinic		
* Hanyangweimin* (vs. *Diyi*)	0.82 (0.55, 1.19)	0.485
* Gutian* (vs. *Diyi*)	1.28 (0.32, 2.23)	0.698
Gender: female (vs. male)	1.64 (1.04, 2.60)	0.034
Age (years)	1.02 (0.98, 1.06)	0.318
Education: primary school and illiterate (vs. junior high school and above)	3.71 (1.94, 7.07)	<0.001
Marital status: non-married (vs. married)*	1.34 (0.87, 2.08)	0.184
Unemployment (vs. employment)	2.28 (1.50, 3.45)	<0.001
Main route of heroin administration: injecting (vs. smoking)	4.47 (2.28, 8.79)	<0.001
Duration of heroin use (years)	1.07 (1.01, 1.12)	0.014
Duration of methadone maintenance therapy (months)	0.99 (0.97, 1.01)	0.415
Dosage of methadone (mg/d)	1.01 (0.99, 1.02)	0.111
Pain	1.89 (1.20, 2.96)	0.006
Depressive symptoms	3.59 (2.18, 5.86)	<0.001

*”Married” includes married and remarried; “non-married” includes never-married, separated, cohabitating, divorced, and widowed.

CI, confidence interval; OR, odds ratio; MMT, methadone maintenance therapy.

## Discussion

To the best of our knowledge, this is the first empirical study in China examining the association between Buddhist belief and suicide risk in patients with heroin dependence. Results from between-group comparison showed a significantly higher level of current suicide risk in Buddhist than non-religious believers. The elevated suicide risk in Buddhist believers was further strengthened by the significant association between Buddhist belief and an increase in the level of current suicide risk after adjusting for potential confounders, that is, the significantly higher current suicide risk in Buddhist than non-religious believers is independent of their demographic and clinical factors (including depressive symptoms). Notably, the strength of the association of suicide risk with Buddhism (OR: 2.98) is close to that with depression (OR: 3.59) and higher than that with pain (OR: 1.89) (**Table 2**). Because the latter two are well-known major risk factors for suicidal behaviors (41, 47–49), Buddhist belief appears to be the other major factor associated with elevated suicide risk in Chinese patients receiving MMT for heroin dependence.

The first code of ethics from the five precepts within Buddhism doctrine is the abstinence from killing living beings, including intentionally ending one's own life by one's own hand, which is strictly prohibited by Buddhism (30, 50). In the Buddhism teaching, suicidal behaviors are regarded as an “unskilled action”, which would cause “*dukkha”* (unsatisfactoriness or suffering) to persons after the self-destructive behaviors (51). Although human life is also full of “*dukkha”* in Buddhism, suicide is useless in escaping from suffering and difficulties, because Buddhism views life and death are a great cycle, and death does not mean the end of life but a beginning of a new cycle. In this case, death by suicide would result in the premature cessation of a valuable human rebirth and trigger another cycle of life with more “*dukkha”* (51, 52). As confirmed by an qualitative study, Buddhism lowers an individual's risk of suicidal ideation *via* its influence on reasons to not attempting suicidal behaviors (53). Due to these reasons, Buddhist belief has been associated with lower risk of suicidal ideation in community-dwelling residents in Vietnam, and with lower risk of suicide attempt in university students of 12 countries (23, 54). However, our study found a positive association between Buddhist belief and elevated suicide risk, which is opposite to the Buddhist perspectives on suicide and findings from the above-mentioned studies.

In China, although people can enjoy the freedom of religious belief, believing in religion is not a common phenomenon in this world's most atheistic country. We therefore consider that the elevated suicide risk in Buddhist believers of MMT patients may result from their social identity pressures. Moreover, unlike religious believers in western countries, the religiosity of religious believers and followers in China is relatively low, including Buddhist believers (55). Many Buddhist believers seldom attend the religious activities unless they are mentally distressed and suffer from suicidal problems, so Buddhist belief may not work in preventing or reducing suicide due to the lack of continuous religious group support. In fact, a very common phenomenon in the Chinese general population is that people often turn to religion when they are emotionally distressed or have suicidal tendency and need to seek help from the religion (55, 56). Because of their own mental health and suicidal problems and their religious help-seeking behaviors, Buddhism would “passively” have a positive association with suicide risk. We believe that this explanation is more likely because the majority of patients with heroin dependence, including those receiving MMT, have a high unmet need for their physical and psychosocial problems (31–33). In addition, the unexpectedly higher prevalence of Buddhist belief in our sample of patients with heroin dependence (10.1%, 61/603) than the Chinese general population (6.8%) (22) seems to support this speculation, because this is not in line with the Buddhist precept of abstaining from intoxicating drinks and drugs. The most possible answer is that some patients turn to Buddhism for seeking help and supports for their physical and psychosocial problems caused by heroin dependence, or even confessing for their past misconducts.

The major limitation of this study is that we did not collect data on reasons for believing in Buddhism and whether Buddhist belief preceded suicidal behaviors. Due to these, we cannot ascertain the causality between Buddhist belief and elevated suicide risk. The second limitation is no assessment of frequency of attending Buddhist activities, which would provide more useful information on the relationship between Buddhist belief and suicide risk. Third, some variables associated with increased suicide risk such as inadequate social support and negative life events were not collected in this study. Studies collecting these data are warranted to clarify the influence of these variables on the association between Buddhist belief and suicide risk in patients receiving MMT. Despite these limitations, the increased suicide risk in Buddhist believers of patients with heroin dependence is an interesting and clinically relevant phenomenon in Chinese MMT settings. Health care providers in Chinese MMT clinics may consider Buddhist belief as a marker or clue of a high current suicide risk of the patients and provide a timely psychiatric assessment and crisis intervention (if necessary) to those at risk of suicide.

## Data Availability Statement

The datasets generated for this study are available on request to the corresponding author.

## Ethics Statement

The studies involving human participants were reviewed and approved by The Institutional Review Board of Wuhan Mental Health Center. The patients/participants provided their written informed consent to participate in this study.

## Author Contributions

J-XH and B-LZ were responsible for the design of the study and interpretation of data, and Y-MX for the manuscript draft, statistical analysis, the data collection, and critical revision of the manuscript. All authors reviewed the data and analysis, revised the manuscript, had full access to all of the data in the study, can take responsibility for the integrity of the data and the accuracy of the data analysis, and had authority over approval of final manuscript version and the decision to submit for publication.

## Funding

This study was funded by the National Social Science Foundation of China (grant number 15CZJ018, J-XH, PI), and Wuhan Health and Family Planning Commission [WX17Q30, Y-MX, PI; WG16A02, B-LZ, PI]. The funders had no role in study design, data collection and analysis, decision to publish, or preparation of the manuscript.

## Conflict of Interest

The authors declare that the research was conducted in the absence of any commercial or financial relationships that could be construed as a potential conflict of interest.
